# Revealing epilepsy type using a computational analysis of interictal EEG

**DOI:** 10.1038/s41598-019-46633-7

**Published:** 2019-07-15

**Authors:** Marinho A. Lopes, Suejen Perani, Siti N. Yaakub, Mark P. Richardson, Marc Goodfellow, John R. Terry

**Affiliations:** 1 Living Systems Institute, University of Exeter, Exeter, EX4 4QD UK; 2 Centre for Biomedical Modelling and Analysis, University of Exeter, Exeter, EX4 4QD UK; 3 EPSRC Centre for Predictive Modelling in Healthcare, University of Exeter, Exeter, EX4 4QD UK; 4 Institute of Psychiatry, Psychology and Neuroscience, King’s College London, London, SE5 8AF UK; 5 King’s College Hospital NHS Foundation Trust, London, SE5 9RS UK

**Keywords:** Diagnostic markers, Diagnostic markers, Epilepsy

## Abstract

Seizure onset in epilepsy can usually be classified as focal or generalized, based on a combination of clinical phenomenology of the seizures, EEG recordings and MRI. This classification may be challenging when seizures and interictal epileptiform discharges are infrequent or discordant, and MRI does not reveal any apparent abnormalities. To address this challenge, we introduce the concept of *Ictogenic Spread* (IS) as a prediction of how pathological electrical activity associated with seizures will propagate throughout a brain network. This measure is defined using a person-specific computer representation of the functional network of the brain, constructed from interictal EEG, combined with a computer model of the transition from background to seizure-like activity within nodes of a distributed network. Applying this method to a dataset comprising scalp EEG from 38 people with epilepsy (17 with genetic generalized epilepsy (GGE), 21 with mesial temporal lobe epilepsy (mTLE)), we find that people with GGE display a higher IS in comparison to those with mTLE. We propose IS as a candidate computational biomarker to classify focal and generalized epilepsy using interictal EEG.

## Introduction

Epilepsy is a neurological disorder characterized by recurrent seizures^1^. According to the International League Against Epilepsy (ILAE), the diagnosis of epilepsy comprises three levels^2^: the identification of seizure type^3^, the classification of epilepsy type^2^, and diagnosis of epilepsy syndrome, if possible. Four seizure-onset patterns are currently recognized: focal, generalized, combined generalized and focal, and unknown^2^. The diagnosis of generalized and focal epilepsy is based on clinical grounds, supported by EEG findings. When there is insufficient information to determine the epilepsy type, the clinician may use the term unknown epilepsy until a more accurate classification may become possible^2^. Classification as generalized or focal epilepsy is important as it has a strong relationship with the potential underlying aetiology and determines the first line of treatment as well as longer-term management options such as surgery. Prognosis depends on the type of epilepsy since generalized epilepsies usually respond better to medication^4^.

Diagnosis of seizure type is primarily based on clinical history of the seizure phenomena. Seizure semiology plays a crucial role in the seizure classification system^3,5^ but may only be considered if clinical seizures are observed. Furthermore, semiology interpretation may vary between neurologists^6^. On the other hand, pathological interictal spikes, spike-waves, and sharp waves (collectively referred to as interictal epileptiform discharges (IEDs)) can be used to support diagnosis, however their sensitivity can be as low as 29% from a first EEG^7^. When detected, IEDs may contribute to diagnosing the seizure type and epilepsy type, with generalized IEDs suggesting a generalized epilepsy, and focal IEDs suggesting a focal epilepsy^7^. IEDs may be absent because they are infrequent or because they originate in deep sources, and therefore might not be visible on the scalp^7^. Recording a seizure onset during EEG often provides robust evidence of focal or generalized epilepsy but is unlikely during a routine 60–90 minute diagnostic clinical EEG.

Since seizures and IEDs are typically rare events, clinical EEG consists largely of apparently normal brain activity (e.g. interictal EEG). In recent years, a growing body of literature has supported the hypothesis that apparently normal EEG may also be informative about possible underlying epilepsy. For example, Larsson *et al*.^8^ showed that the EEG power spectrum from people with epilepsy has a shift in the peak of alpha power towards lower frequencies compared to a group of people without epilepsy. Horstmann *et al*.^9^ found a tendency for functional networks inferred from people with epilepsy to exhibit greater clustering and therefore more regularity than controls. Similarly, Quraan *et al*.^10^ observed that functional networks from people with epilepsy deviated from small-world network structures found in healthy controls. Van Diessen *et al*.^11^ also used resting-state EEG to build a multivariable decision tree based on functional network properties that was capable of distinguishing children with focal epilepsy from healthy children. In our prior work^12^, we revealed a brain network endophenotype in patients with idiopathic generalized epilepsy (IGE) and their relatives compared to healthy controls. In this study, brain networks were also constructed from resting-state scalp EEG, and an elevated average number of connections in networks from individuals with IGE and their relatives were found, compared to healthy controls^12^. Furthermore, it has also been shown that directed functional networks inferred from interictal high-density scalp EEG may be informative of cognitive deficits in TLE^13^ and may be used to diagnose TLE even in the absence of interictal spikes^14^. Also, van Diessen *et al*.^15^ used interictal EEG from drug-naïve children with newly diagnosed focal and generalized epilepsy and controls to show that network alterations could be identified at an early stage of focal epilepsy. More recently, Verhoeven *et al*.^16^ implemented an automated diagnosis tool to lateralize TLE based on apparently normal EEG. Moving from these observational studies, we developed a framework to study the mechanisms by which network alterations lead to pathological activity^17^. Here we showed that a computational biomarker based on clinical resting-state EEG could support diagnosis of generalized epilepsies^17,18^.

We have recently studied the propensity of different synthetic network topologies to generate seizure-like activity^19^. We quantified this propensity in terms of *Brain Network Ictogenicity* (BNI), i.e. the average time that each network node spends in the seizure state^12,19,20^, and found that BNI depends on the network structure (Fig. 4 in ref.^19^). We observed that some network topologies were more prone to widespread seizure emergence across the network compared to others and this was characterized by specific BNI features. In the current study, we therefore aim to test whether properties of the BNI are capable of distinguishing between focal and generalized epilepsy, based on a dynamic network model informed by functional networks inferred from scalp EEG.

## Results

### Quantification of Ictogenic spread (IS)

We studied a total of 38 adult individuals with epilepsy: 17 with GGE (9 female, mean age 25.8 years) and 21 with mTLE (10 female, mean age 40.5 years). Participants were asked to rest with their eyes closed while scalp EEG was recorded with a 64-channel MR-compatible cap at a sampling rate of 5 kHz (see Methods). The data was pre-processed and continuous 20 second artifact-free segments were extracted from the recordings. We found 21 ± 14 segments per individual in the GGE dataset and 12 ± 8 segments in the mTLE dataset. A total number of 623 segments were considered. We focused our analysis in two different frequency bands, low-alpha (6–9 Hz) and broadband (1–25 Hz). For each frequency band, we extracted a total number of 623 functional networks using the Phase Locking Factor (PLF)^21–23^ each derived from a 20 second EEG segment (see Methods). Each functional network was then studied using a phenomenological model of seizure transitions to characterize its propensity to generate generalized or focal epileptiform dynamics. To quantify this propensity, we measured BNI as a function of the global scaling *K* of the coupling coefficients computed from the functional connectivity (see Methods).

**Figure 1:**
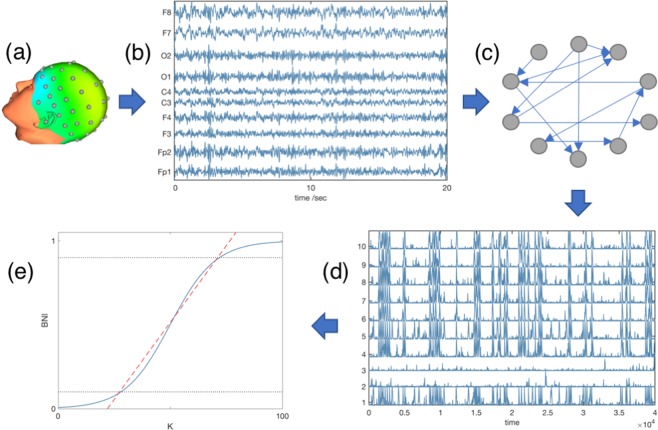
Scheme of the data analysis procedure. (**a**) Electrodes were placed on the scalp of 17 individuals suffering from genetic generalized epilepsy and 21 individuals with mesial temporal lobe epilepsy. (**b**) EEG signals were recorded and several 20 second artifact-free segments were selected per individual. This panel displays 10 EEG channels for representative purposes though in our analysis we considered 64 EEG channels. (**c**) Functional networks were constructed from the EEG signals. (**d**) Model-generated data was obtained by placing a mathematical model on each node of the functional networks. (**e**) The Brain Network Ictogenicity (BNI) as a function of the global scaling factor *K* was measured from the model-generated data. The ictogenic spread is defined as the average slope of this curve between BNI = 0.1 and BNI = 0.9 (the dashed red line).

**Figure 2:**
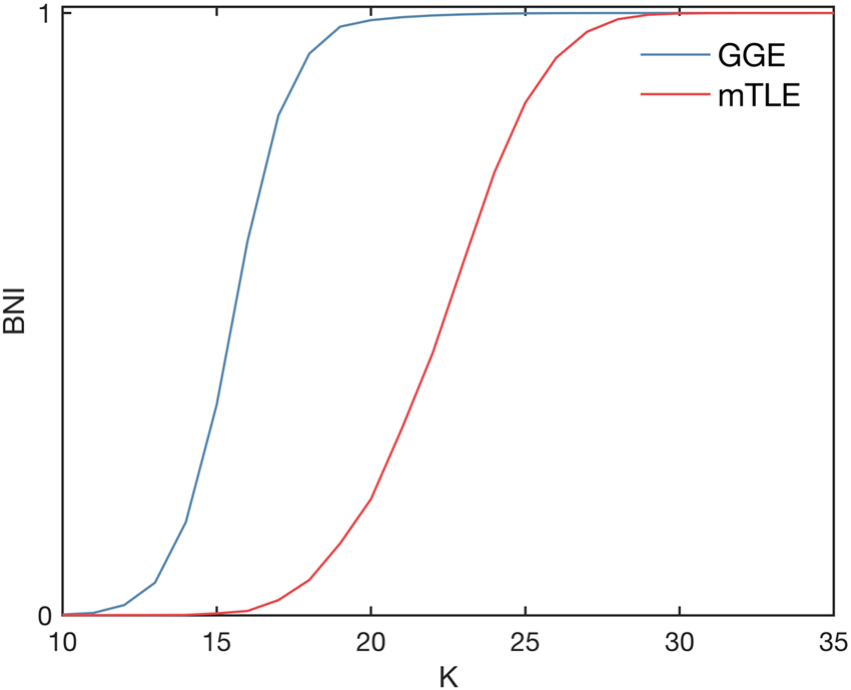
Two representative Brain Network Ictogenicity (BNI) curves as a function of the global scaling factor *K* computed from two functional networks. The blue curve corresponds to an individual with genetic generalized epilepsy, and the red curve to an individual with mesial temporal lobe epilepsy. The standard error of the BNI is not represented as it is almost indistinguishable given the scale of the figure.

Figure 1 summarizes our analysis: we inferred a functional network from each 20 second artifact-free segment, then used a mathematical model to study the propensity of the network to generate focal or generalized dynamics by calculating the IS. Thus, for each individual we obtained a distribution of IS values. Figure 2 shows two representative BNI curves, one from an individual with GGE and another from an individual with mTLE. We observe that the GGE curve is steeper than the mTLE curve.

### IS comparison using all data

**Figure 3:**
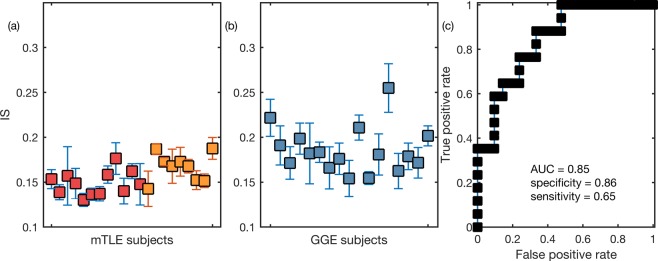
Ictogenic Spread (IS) of the genetic generalized epilepsy (GGE) and mesial temporal lobe epilepsy (mTLE) individuals. Each marker in panels (a and b) represents the mean IS of a single individual and the error bars account for the variability of IS measured across different functional networks of different EEG segments band-pass filtered between 1 and 25 Hz. Panel (a) and (b) show the IS of mTLE and GGE subjects, respectively. In panel (a), the red markers identify left mTLE individuals, whist the orange markers correspond to right mTLE individuals. The GGE group has a larger IS than the mTLE group (*p* < 0.001, Mann–Whitney U test with Bonferroni-Holm correction for multiple comparisons in the two frequency bands). Panel (c) exhibits the receiver operating characteristic (ROC) curve for genetic generalized epilepsy versus mTLE subjects using the IS as a classifier. The area under the curve (AUC) is 0.85, and the optimal specificity and sensitivity are 0.86 and 0.65, respectively.

Our hypothesis is that the curves computed from the functional networks of the GGE group should have a steeper slope compared to the curves of the mTLE group, i.e., a larger IS. Figure 3(a,b) show the IS of each individual using the functional networks inferred in the 1–25 Hz frequency band. Note that since we have multiple functional networks per individual, each marker corresponds to the average IS across all functional networks of a single individual, and the error bars correspond to the standard deviation of the IS. We find that the GGE group has higher values of IS relative to the mTLE group (*p* < 0.001, Mann–Whitney U test with Bonferroni-Holm correction for two comparisons in the two frequency bands). The AUC of the ROC curve in Fig. 3(c) is 0.85. We found similar results in the 6–9 Hz frequency band (*p* = 0.002, same statistical test as above), with a slightly lower AUC = 0.78. Figure 3(a) further indicates that right mTLE individuals have higher IS compared to left mTLE (*p* = 0.02, same statistical test as above). We observe the same relationship in the low-alpha frequency band (*p* = 0.02, same statistical test as above).

### IS comparison using an equal number of segments per individual

Given that different individuals had a different number of 20 second artifact-free segments, we repeated the same comparison in the broadband using 3 segments for each and every individual (i.e. the smallest number of segments in any individual). The 3 segments were randomly selected for individuals who had more segments. We also found higher IS in the GGE group compared to the mTLE group (*p* < 0.001, same statistical test as above, and AUC = 0.83).

### IS comparison in age and gender matched individuals

We further compared the IS in a subset of 14 GGE and 14 mTLE individuals age and gender matched and found similar results (see Supplementary Fig. S1). Finally, we assessed whether epilepsy duration could be the reason why mTLE individuals display lower IS compared to GGE individuals given that the two groups have on average different epilepsy durations. Supplementary Figure S2 shows that IS does not correlate with epilepsy duration within the mTLE and GGE groups and thus could not account for the difference between groups. Additionally, whilst all mTLE individuals had ongoing seizures, some of the GGE individuals were seizure-free. We therefore sought to understand whether this difference could account for the difference in IS between the two groups (see Supplementary Fig. S2(b)). We performed a Mann–Whitney U test to compare IS from seizure-free and non-seizure free individuals with GGE and found no statistical difference between the two groups.

### IS comparison using a single segment per individual

We also estimated the chance of finding these results from a single 20 second segment per individual in the 1–25 Hz frequency band to examine whether a single segment is enough to observe an equivalent group classification as using all the data. We repeated the statistical analysis 1000 times using only one randomly selected segment per individual. We found a 99% chance of observing a statistically significant higher IS in the GGE group compared to the TLE group, but just an 11 % chance of finding an equal or higher AUC compared to the case with all the segments. The average AUC was 0.78.

### IS comparison restricted to 19-channel data

Finally, since clinical EEG is most often recorded using a 19-channel system, we repeated our analysis using data only recorded from the standard 19 channels: Fp1, Fp2, F7, F3, Fz, F4, F8, T7, C3, Cz, C4, T8, P7, P3, Pz, P4, P8, O1, and O2. Thus, instead of the previous networks with 64 nodes, we considered networks comprising only 19 nodes. Note that as we used a bivariate method to construct the functional networks, we did not have to compute the functional networks again, instead we kept the nodes of interest and respective pairwise functional connections. We restricted our evaluation to the above best performing frequency band, 1–25 Hz. Again, we found higher IS in the GGE group compared to the mTLE group (*p* = 0.005, same statistical test as above, and AUC = 0.75; see Supplementary Fig. S3). We also tested the robustness of this finding when using only one 20 second segment per individual and found a 76% chance of observing a statistically significant result and a 22% chance of finding an equal or higher AUC (the average AUC was 0.70). This 22% chance is higher than the one observed using the 64-channel data (11%) because the AUC based on the 19-channel confined data is lower than the one found using the 64-channel data.

## Discussion

In this study, we explored whether generalized and focal epilepsies can be differentiated using interictal EEG. This is an important question because, in the clinical setting, EEG in people with suspected epilepsy is typically free of discharges and other epileptiform abnormalities. Therefore, the discovery of biomarkers in interictal EEG would improve the clinical utility of EEG. We considered a dataset of scalp EEG collected from 38 individuals with epilepsy, 17 with GGE, and 21 with mTLE. We inferred functional networks from interictal EEG using the PLF. In order to distinguish the functional networks between the two groups, we introduced the Ictogenic Spread (IS), which, as articulated in the Results, quantifies the propensity of a network to generate focal or generalized seizures *in silico*. To account for the fact that functional networks are time-dependent and therefore model predictions may vary depending on the considered network, we used multiple functional networks per individual. The GGE group exhibited a higher IS than the mTLE group (see Fig. 3). These findings are in line with our previous theoretical results, in which we showed that networks with focal ictogenic nodes displayed a more gradual increase of BNI as a function of global coupling (i.e. lower IS) compared to other networks without such focal ictogenic drivers^19^. We further showed that the result was significant using only one 20 second segment of EEG data per individual or even standard 19-channel EEG instead of 64-channel EEG data, although with lower classification success (see Supplementary Fig. S3).

There are a number of possible confounding factors that could account for the observed results. First, the mTLE group is on average older than the GGE group. To address this, we compared a subset of 28 individuals age (and gender) matched and found equivalent results (see Supplementary Fig. S1). Second, the mTLE group has on average a longer epilepsy duration than the GGE group. However, we do not observe a correlation between IS and epilepsy duration within the mTLE and GGE groups separately (see Supplementary Fig. S2). Third, some of the GGE individuals obtained seizure control under medication, whilst all mTLE individuals were not seizure-free. We thus compared the IS between non-seizure-free and seizure-free individuals within the GGE group and found no statistical difference (see Supplementary Fig. S2(b)). We therefore suggest that these factors do not influence the IS. Nevertheless, we acknowledge that the framework may be improved in a number of ways. In particular, there are multiple possible methods to infer functional networks from scalp EEG^24^. For example, it has been shown that an orthogonalization of source reconstructed signals may offer superior predictions of functional connectivity^25,26^. Different methods may extract different information from the EEG data and therefore model predictions may vary upon the choice of functional network measure. Thus, future studies should compare the IS using other methods to construct functional networks. Also, here we chose to examine only two frequency bands (see Methods), however, other frequency bands may be more informative. Finally, the employed model may be over-simplistic for the purpose of epilepsy classification. A more sophisticated model could enable fitting other data properties which in turn could lead to better predictions. Such analysis may lead to an optimized framework with superior classification performance. However, a comprehensive comparison of all these methodological choices will demand a much larger dataset than the one used in this study.

It has been demonstrated that transcranial magnetic stimulation (TMS) is capable of unveiling differences between generalized and focal epilepsy^27^. In particular, it was shown that individuals with idiopathic generalized epilepsy (IGE) required a stronger TMS to recruit intracortical inhibition compared to those with focal epilepsy^27^. We suggest that such difference may be a consequence of different underlying network mechanisms. Based on our results, we further suggest that these mechanisms are expressed in functional networks inferred from interictal EEG.

Interestingly, within the mTLE group we found that individuals with right mTLE exhibited higher IS than individuals with left mTLE. This result is in agreement with previous diffusion tensor imaging studies that have shown that left and right TLE are not symmetric pathologies^28,29^. Left TLE was associated with a much more pronounced reduction of fractional anisotropy in the ipsilateral temporal lobe compared to controls^29^. This more marked structural alteration in left TLE may explain why we find left mTLE with lower IS relative to right mTLE.

In this study, we considered individuals on antiepileptic drugs. We aim in future work to study newly diagnosed and untreated individuals. This will allow us to control for the potential effect of a prolonged pathology and the effect of medication on brain networks. Furthermore, future studies should also explore whether our framework based on the concept of the IS may also be useful in distinguishing structural networks inferred from individuals with generalized and focal epilepsy. Also, taking into account that the IS was capable of distinguishing left and right mTLE, future work should aim to further develop the computational framework to test whether it is capable of localizing focal epilepsies. This framework may then be compared to other recent approaches which have shown promise at the group level in identifying hemispheric abnormalities in cohorts of left and right focal epilepsies based on interictal scalp EEG^30^.

The methods proposed here are one instance of a more general framework that has been developed in recent years to study brain networks based on simulations of brain activity^31–34^. EEG or other data modalities allow us to infer a network representation of the brain, whose properties can then be examined by using a model of brain dynamics. In the context of epilepsy, this framework has been used to study epilepsy diagnosis^17,18^, epilepsy surgery^34–36^, seizure propagation^37^, and epileptogenesis in idiopathic generalized epilepsy^38^ using different data modalities. Here, we further extended the framework to differentiate between focal and generalized epilepsy. Contrarily to previous studies focused on network differences^9–12,15^, the framework employed in this study has the potential of uncovering mechanistic insights of the underlying pathologies. It is important to note that we are not simply distinguishing mTLE from GGE individuals. Instead, we predicted that GGE should present higher IS values than mTLE due to the fact that the underlying brain dynamics *in silico* that are supported by GGE functional networks are expected to be more generalized than the dynamics supported by mTLE functional networks. Our results thus suggest that even apparently normal scalp EEG hold information about the pathophysiological features of epilepsy type.

In concluding: at present the classification of epilepsy type is mainly based on the clinical observation of seizures and IEDs^2^. In this study, we showed that interictal EEG can be informative and support the classification of epilepsy type as either focal or generalized. Such methods that rely only on interictal EEG may offer additional clinical value, removing the reliance on observing seizures or IEDs as well as reducing the need for prolonged monitoring.

## Methods

### Recruitment and selection of participants

GGE individuals were recruited from seizure clinics across London, and mTLE individuals were recruited from outpatient epilepsy and neurology clinics in south London. The diagnosis of mTLE or GGE was made by an epilepsy specialist on the basis of clinical evaluation including seizure history, scalp EEG recordings, and conventional clinical MRI reported by experienced neuroradiologists. All patients were on anti-epileptic drugs (AEDs) at the time of the study. We excluded patients with history of any neurological condition other than epilepsy. Five GGE individuals were seizure-free from about 6 months after diagnosis, whereas all other individuals were not. A full list of the demographic characteristics of the patients is available in Tables 1 and 2. In accordance with approved guidelines, the study was conducted at the National Institute for Health Research/Wellcome Trust King’s Clinical Research Facility at King’s College Hospital and approved by the Riverside Research Ethics Committee (REC approval number 12/LO/2006), and the Bromley REC (14/LO/0193). Written informed consent was obtained from all participants after all procedures were fully explained.

**Table 1:** Clinical characteristics of the individuals with genetic generalized epilepsy.

ID	Age	Gender	Syndrome	Seizure freedom	Epilepsy duration	Medication	# of 20 sec segments
GGE01	22	M	JME	No	1	VPA	57
GGE02	14	F	JME	No	1	LMT	37
GGE03	20	F	JME	Yes*	1	LEV	16
GGE04	36	M	JME	No	20	VPA	31
GGE05	18	F	GTCSO	No	1	unknown	20
GGE06	37	F	JME	Yes*	1	LMT	12
GGE07	26	F	GTCSO	Yes*	1	LMT	24
GGE08	18	F	JME	Yes*	1	LMT	15
GGE09	22	F	GTCSO	No	4	LMT	37
GGE10	39	M	GTCSO	No	11	LEV	12
GGE11	40	M	JME	No	32	VPA	5
GGE12	21	M	JME	No	14	VPA	14
GGE13	20	M	JME	No	4	VPA, LEV	26
GGE14	22	F	JME	No	7	LMT, LEV	33
GGE15	30	F	JME	No	23	VPA, LEV, PER	9
GGE16	40	M	JME	No	25	VPA	13
GGE17	14	M	JME	Yes*	1	VPA	3

Age and epilepsy duration is in years, M = male, F = female, JME = juvenile myoclonic epilepsy, GTCSO = generalized tonic clonic seizure only, VPA = valproate, LEV = levetiracetam, LMT = lamotrigine, PER = perampanel. *Seizure-free individuals had not experienced seizures from about 6 months after diagnosis.

**Table 2:** Clinical characteristics of the individuals with mTLE.

ID	Age	Gender	Syndrome	Seizure freedom	Epilepsy duration	Medication	# of 20 sec segments
TLE01	41	F	Right mTLE	No	17	LMT, LEV, PER, CLB	4
TLE02	43	F	Left mTLE	No	23	CAR, LMT	3
TLE03	57	F	Left mTLE	No	52	LEV, CIT	15
TLE04	22	M	Left mTLE	No	6	CAR	14
TLE05	34	M	Right mTLE	No	23	PHB, VPA, OLA, CIT	3
TLE05	52	F	Left mTLE	No	37	LAC, CIT, LOR	6
TLE06	51	F	Left mTLE	No	20	LAC	4
TLE08	31	M	Right mTLE	No	6	CAR, LEV, CLB	8
TLE09	48	M	Right mTLE	No	15	LEV, TOP	12
TLE10	31	M	Right mTLE	No	10	LEV, ZON, CLN	23
TLE11	58	F	Left mTLE	No	11	TOP, CLB	10
TLE12	24	M	Right mTLE	No	2	CAR	25
TLE13	25	M	Left mTLE	No	2	VPA, TOP	5
TLE14	43	F	Left mTLE	No	3	CAR	16
TLE15	23	M	Right mTLE	No	1	ZON	23
TLE16	47	M	Left mTLE	No	32	CAR	23
TLE17	57	M	Right mTLE	No	32	LMT	5
TLE18	37	F	Left mTLE	No	10	CAR	14
TLE19	31	F	Left mTLE	No	9	LEV	17
TLE20	44	F	Left mTLE	No	43	CAR, CLB	22
TLE21	52	M	Right mTLE	No	27	LMT, PER	7

Age is in years, M = male, F = female, LEV = levetiracetam, LMT = lamotrigine, PER = perampanel, CLB = clobazam, CAR = carbamezapine, PHB = phenobarbitone, VPA=valproate, OLA=olanzapine, CIT=citalopram, ZON=zonisamide, LAC=lacosamide, LOR=lorazepam, CLN=clonazepam, TOP=topiramate.

### EEG acquisition

Scalp EEG was recorded with a 64-channel MR-compatible cap (BrainAmp MR plus, Brain Products, Gilching, Germany). We used the cap’s standard montage: reference channel between Fz and Cz channels, and the ground channel between Fz and Fpz. EEG data were band-pass filtered at 0.016 Hz–1 kHz, with 16-bit digitalization (0.05 mV resolution) at a sampling rate of 5 kHz. EEG was recorded during echo-planar imaging (EPI) in a General Electric 3.0 Tesla MRI scanner (GE Discovery MR750, General Electric Healthcare Systems, Chicago, USA). During the acquisition, participants were asked to rest with their eyes closed for 2 fMRI sessions of 10 minutes each. These in-scanner data contained considerably more suitable epochs for analysis than a typical 10–20 clinical EEG, hence these data were preferred for this study.

### EEG pre-processing

MR gradient and pulse-related artefacts were removed off-line from the EEG recorded inside the MRI using the template artefact subtraction method^39,40^ implemented in BrainVision Analyser (version 2.0, Brain Products, Germany). The EEG recordings were then reviewed by SP, and artefact-free channels were identified. Since interpreting EEG is a subjective task^41^, we further identified artefact-free data using TAPEEG, a fully automated toolbox for resting-state EEG detection^42^, and considered only the data classified as artefact-free independently by both SP and TAPEEG. We then extracted continuous 20 second artifact-free segments from the recordings. The data were re-referenced to the average of all artifact-free segments, and down-sampled to 250 Hz (Matlab function resample, which uses a polyphase anti-aliasing filter).

The pre-processed data were analyzed in two different frequency bands, low-alpha (6–9 Hz) and broadband (1–25 Hz). We chose the low alpha band given previous evidence showing that functional networks inferred from this frequency band were capable of distinguishing between people suffering from generalized epilepsy and healthy controls^17,18^. The broadband was considered in order to encapsulate the traditional clinical frequency bands (delta, theta, alpha, and most of beta^43^) and explore more broadly potential features in the epochs, while avoiding high frequencies which can embed muscle electrical activity^44^. A fourth-order Butterworth filter was applied with forward and backward filtering to minimize phase distortions.

### Inferring functional networks from EEG

The functional networks were inferred using a method based on the Phase Locking Factor (PLF)^21–23^ as previously described in refs.^17,18^. Again, we chose to use this functional network measure due to its demonstrated capability to distinguish generalized epilepsy from healthy controls^17,18^. Electrode locations were considered as nodes and PLF values as connectivity weights. For each pair of nodes *i* and *j*, we found the PLF:

$$PL{F}_{ij}=\,\frac{1}{{N}_{s}}|\sum _{k=1}^{{N}_{s}}{e}^{i{\rm{\Delta }}{\varphi }_{ij}({t}_{k})}|$$

where *N*_*s*_ is the number of samples, and Δ*ϕ*_*ij*_(*t*_*k*_) is the instantaneous phase difference between the signals recorded from electrodes *i* and *j* at time *t*_*k*_. The phase differences were computed using the Hilbert transform on the down-sampled, filtered signals. We also found the average time-lag *τ*_*ij*_ between the two signals,

$${\tau }_{ij}=arg(\sum _{k=1}^{{N}_{s}}{e}^{i{\rm{\Delta }}{\varphi }_{ij}({t}_{k})})$$

Nodes *i* and *j* were considered connected if *PLF*_*ij*_ > 0 and *τ*_*ij*_ > 0 with connection weight *PLF*_*ij*_. We only considered non-zero time-lag PLF to avoid possibly artefactual connections due to volume conduction^24^. We further excluded spurious connections due to finite length time-series data. We generated 99 surrogates from the original EEG signals using the iterative amplitude-adjusted Fourier transform (IAAFT) with 10 iterations^45,46^. We rejected connections if their weights *PLF*_*ij*_ did not exceed the 95% significance level compared to the same connection weights as computed from the surrogates. This method yielded a directed weighted functional network *a*_*ij*_ from each data segment.

### Mathematical model

We studied the inherent propensity of a functional network to generate focal or generalized dynamics using a mathematical model at each network node^17,19,20,34^. The brain activity at node *i* was represented by a phase oscillator *θ*_*i*_. We defined a ‘resting state’ as a phase close to a fixed stable phase *θ*^(*s*)^ and an ‘oscillatory state’ as a rotating phase. The resting state represented normal brain activity, whereas the oscillatory state depicted seizure-like activity. The phase oscillator obeyed the following ODE:

$${\dot{\theta }}_{i}=(1-\,\cos \,{\theta }_{i})+(1+\,\cos \,{\theta }_{i}){I}_{i}(t),$$

where *I*_*i*_(*t*) was the input current of node *i*. The magnitude of the current determined whether a phase oscillator was at rest (*I*_*i*_ < 0), or oscillating (*I*_*i*_ > 0). The boundary between the two states corresponds to a saddle-node on invariant circle (SNIC) bifurcation. This simple model has been shown to be a useful and reliable proxy of a more complex and biophysical meaningful model of epileptiform dynamics^19^. We assumed equivalence between nodes when in isolation (*I*_*i*_(*t*) = *I*_0_) and consequently, the same steady state *θ*^(*s*)^ for all nodes, which was obtained from setting ${\dot{\theta }}_{i}=0$,

$${\theta }^{(s)}=-\,{\rm{Re}}\{{\cos }^{-1}(\frac{1+{I}_{0}}{1-{I}_{0}})\}.$$

At *I*_0_ < 0, there are two fixed points, one stable (*θ*^(*s*)^), and one unstable (−*θ*^(*s*)^). We took the real part so that *θ*^(*s*)^ = 0 at *I*_0_ > 0.

In general, the input current *I*_*i*_(*t*) encompassed noisy inputs and the interaction with the other nodes:

$${I}_{i}(t)={I}_{0}+{\xi }^{(i)}(t)+\frac{K}{N}\sum _{j\ne i}{a}_{ji}[1-\,\cos ({\theta }_{j}-{\theta }^{(s)})],$$

where *I*_0_ + *ξ*^(*i*)^(*t*) is noise, *N* is the number of nodes, *a*_*ji*_ is *j*, *i*^th^ entry of the adjacency matrix that encodes the functional network, *K* is a global scaling factor of the functional network, and *θ*^(*s*)^ is the steady state of the in-neighbor *j*. The noisy inputs represented signals from other areas of the brain outside of the functional network under consideration, which we assumed to follow a Gaussian distribution (with mean *I*_0_ and variance *σ*^2^). Each node received independent noise,

$${\langle \xi }^{(i)}(t){\xi }^{(j)}({t}^{{\rm{^{\prime} }}})\rangle ={\sigma }^{2}{\delta }_{i,j}\delta (t-{t}^{{\rm{^{\prime} }}}).$$

The multiplier [1 − cos(*θ*_*j*_ − *θ*^(*s*)^)] defined the output of node *j* which was an input to node *i* if there was a directed connection from node *j* to *i*, i.e. *a*_*ji*_ > 0. If node *j* was in the resting state, *θ*_*j*_ ≈ *θ*^(*s*)^, then its output was approximately zero, whereas when it was oscillating it periodically reached its maximum output at *θ*^(*s*)^ + *π*.

The model has three free parameters, *I*_0_, *σ*, and *K*. We used *I*_0_ = −1.2 and *σ* = 0.6 according to ref.^19^. Given that we aim to characterize the role of the network on the emergence of seizure-like activity, these parameters ensure that nodes are typically in the resting state and the transition to seizure-like activity is essentially a function of network interactions. Different choices of *I*_0_ and *σ* are not expected to qualitatively change our results^19^. Although the model has previously been used to study functional networks inferred from intracranial EEG and artificial networks^19,47,48^, it can be used to examine networks constructed from other data modalities.

### Ictogenic spread

The purpose of the mathematical model was to measure the propensity of a given functional network to generate focal or generalized seizure dynamics *in silico*. We quantified the model-generated dynamics using the concept of *Brain Network Ictogenicity* (BNI)^12,19,20,34^, which is the average fraction of time that nodes spent in the oscillatory state:

$${\rm{BNI}}=\frac{1}{N}\sum _{i}\frac{{t}_{sz}^{(i)}}{T}$$

where ${t}_{sz}^{(i)}$ is the time that node *i* spent in the oscillatory state during a total simulation time *T*. We used *T* = 4 × 10^6^ time steps and the oscillatory state was defined as any activity larger than a threshold as described in Lopes *et al*.^19^. This time ${t}_{sz}^{(i)}$ depends on the global scaling factor *K*, and so does the BNI.

We have previously shown that BNI changes according to the global connectivity strength *K*^19,34^. Since BNI is a measure of the spiking activity across the network, a sharp transition in BNI over *K* means that for low *K* there is no spiking across the network and at some critical *K* there is a switch into all nodes spiking, i.e. generalised activity. In contrast, a slower transition means that by changing *K* there is a more gradual recruitment of nodes into spiking, implying that some nodes spike before others, i.e. focal dynamics. Thus, we hypothesize that if functional networks from people with epilepsy underpin the emergence of generalized and focal dynamics, then those derived from people with GGE should be characterized by a steeper BNI curve relative to functional networks from people with mTLE. We therefore introduce a quantity called *Ictogenic Spread* (IS) which is the average slope of the BNI curve as a function of *K*.

In practice, we computed BNI for a number of different *K*_*i*_ values such that BNI would vary between 0.1 and 0.9 (we used about 40 *K*_*i*_ values), found the slope between consecutive points, and averaged all slopes:

$${\rm{IS}}=\frac{{\rm{BNI}}({K}_{i+1})-{\rm{BNI}}({K}_{i})}{{K}_{i+1}-{K}_{i}},\,{\rm{at}}\,0.1 < {\rm{BNI}}(K) < \mathrm{0.9.}$$

Note that by studying BNI as function of *K* we avoided an arbitrary choice of this parameter which scales the network influence on emerging dynamics^47^.

## Supplementary information

Supplementary Information

## Data Availability

All materials (functional networks and code) are available upon request (contact m.lopes@exeter.ac.uk).

## References

[1] Fisher RS (2005). Epileptic seizures and epilepsy: definitions proposed by the International League Against Epilepsy (ILAE) and the International Bureau for Epilepsy (IBE). Epilepsia.

[2] Scheffer IE (2017). ILAE classification of the epilepsies: position paper of the ILAE commission for classification and terminology. Epilepsia.

[3] Fisher RS (2017). Operational classification of seizure types by the International League Against Epilepsy: Position Paper of the ILAE Commission for Classification and Terminology. Epilepsia.

[4] Chen Z, Brodie MJ, Liew D, Kwan P (2018). Treatment outcomes in patients with newly diagnosed epilepsy treated with established and new antiepileptic drugs: a 30-year longitudinal cohort study. JAMA Neurol..

[5] Noachtar S, Peters AS (2009). Semiology of epileptic seizures: a critical review. Epilepsy Behav..

[6] Benbir G, Demiray DY, Delil S, Yeni N (2013). Interobserver variability of seizure semiology between two neurologist and caregivers. Seizure.

[7] Pillai J, Sperling MR (2006). Interictal EEG and the diagnosis of epilepsy. Epilepsia.

[8] Larsson PG, Kostov H (2005). Lower frequency variability in the alpha activity in EEG among patients with epilepsy. Clin. Neurophysiol..

[9] Horstmann MT (2010). State dependent properties of epileptic brain networks: comparative graph–theoretical analyses of simultaneously recorded EEG and MEG. Clin. Neurophysiol..

[10] Quraan MA, McCormick C, Cohn M, Valiante TA, McAndrews MP (2013). Altered resting state brain dynamics in temporal lobe epilepsy can be observed in spectral power, functional connectivity and graph theory metrics. PLoS One.

[11] Van Diessen E, Otte WM, Braun KP, Stam CJ, Jansen FE (2013). Improved diagnosis in children with partial epilepsy using a multivariable prediction model based on EEG network characteristics. PLoS One.

[12] Chowdhury FA (2014). Revealing a brain network endophenotype in families with idiopathic generalised epilepsy. PLoS One.

[13] Coito A (2015). Dynamic directed interictal connectivity in left and right temporal lobe epilepsy. Epilepsia.

[14] Coito A (2016). Altered directed functional connectivity in temporal lobe epilepsy in the absence of interictal spikes: a high density EEG study. Epilepsia.

[15] van Diessen E, Otte WM, Stam CJ, Braun KP, Jansen FE (2016). Electroencephalography based functional networks in newly diagnosed childhood epilepsies. Clin. Neurophysiol..

[16] Verhoeven T (2018). Automated diagnosis of temporal lobe epilepsy in the absence of interictal spikes. Neuroimage Clin..

[17] Schmidt H, Petkov G, Richardson MP, Terry JR (2014). Dynamics on networks: the role of local dynamics and global networks on the emergence of hypersynchronous neural activity. PLoS Comput. Biol..

[18] Schmidt H (2016). A computational biomarker of idiopathic generalized epilepsy from resting state EEG. Epilepsia.

[19] Lopes MA (2017). An optimal strategy for epilepsy surgery: Disruption of the rich-club. PLoS Comput. Biol..

[20] Petkov G, Goodfellow M, Richardson MP, Terry JR (2014). A critical role for network structure in seizure onset: a computational modeling approach. Front. Neurol..

[21] Tass P (1998). Detection of n: m phase locking from noisy data: application to magnetoencephalography. Phys. Rev. Lett..

[22] Lachaux JP, Rodriguez E, Martinerie J, Varela FJ (1999). Measuring phase synchrony in brain signals. Hum. Brain Mapp..

[23] Mormann F, Lehnertz K, David P, Elger CE (2000). Mean phase coherence as a measure for phase synchronization and its application to the EEG of epilepsy patients. Physica D.

[24] Bastos AM, Schoffelen JM (2016). A tutorial review of functional connectivity analysis methods and their interpretational pitfalls. Front. Syst. Neurosci..

[25] Brookes MJ, Woolrich MW, Barnes GR (2012). Measuring functional connectivity in MEG: a multivariate approach insensitive to linear source leakage. Neuroimage.

[26] Hipp JF, Hawellek DJ, Corbetta M, Siegel M, Engel AK (2012). Large-scale cortical correlation structure of spontaneous oscillatory activity. Nat. Neurosci..

[27] Klimpe S, Behrang-Nia M, Bott MC, Werhahn KJ (2009). Recruitment of motor cortex inhibition differentiates between generalized and focal epilepsy. Epilepsy Res..

[28] Ahmadi ME (2009). Side matters: diffusion tensor imaging tractography in left and right temporal lobe epilepsy. AJNR Am. J. Neuroradiol..

[29] Besson P (2014). Structural connectivity differences in left and right temporal lobe epilepsy. NeuroImage.

[30] Woldman, W. *et al*. Dynamic network properties of the interictal brain determine whether seizures appear focal or generalised. *bioRxiv* 576785 (2019). 10.1038/s41598-020-63430-9 PMC7184577 32341399

[31] Honey CJ, Sporns O (2008). Dynamical consequences of lesions in cortical networks. Hum. Brain Mapp..

[32] Alstott J (2009). Modeling the impact of lesions in the human brain. PLoS Comput. Biol..

[33] Sanz Leon P (2013). The Virtual Brain: a simulator of primate brain network dynamics. Front. Neuroinform..

[34] Goodfellow M (2016). Estimation of brain network ictogenicity predicts outcome from epilepsy surgery. Sci. Rep..

[35] Sinha N (2017). Predicting neurosurgical outcomes in focal epilepsy patients using computational modelling. Brain.

[36] Jirsa VK (2017). The virtual epileptic patient: individualized whole-brain models of epilepsy spread. Neuroimage.

[37] Proix T, Jirsa VK, Bartolomei F, Guye M, Truccolo W (2018). Predicting the spatiotemporal diversity of seizure propagation and termination in human focal epilepsy. Nat. Commun..

[38] Sinha N (2019). Computer modelling of connectivity change suggests epileptogenesis mechanisms in idiopathic generalised epilepsy. Neuroimage Clin..

[39] Allen PJ, Polizzi G, Krakow K, Fish DR, Lemieux L (1998). Identification of EEG events in the MR scanner: the problem of pulse artifact and a method for its subtraction. NeuroImage.

[40] Allen PJ, Josephs O, Turner R (2000). A method for removing imaging artifact from continuous EEG recorded during functional MRI. NeuroImage.

[41] Azuma H (2003). An intervention to improve the interrater reliability of clinical EEG interpretations. Psychiatry Clin. Neurosci..

[42] Hatz F (2015). Reliability of fully automated versus visually controlled pre-and post-processing of resting-state EEG. Clin. Neurophysiol..

[43] Buzsaki, G. *Rhythms of the Brain* (Oxford University Press, 2006).

[44] Whitham EM (2007). Scalp electrical recording during paralysis: quantitative evidence that EEG frequencies above 20 Hz are contaminated by EMG. Clin. Neurophysiol..

[45] Schreiber T, Schmitz A (1996). Improved surrogate data for nonlinearity tests. Phys. Rev. Lett..

[46] Schreiber T, Schmitz A (2000). Surrogate time series. Physica D.

[47] Lopes MA (2018). Elevated ictal Brain network ictogenicity enables Prediction of Optimal seizure control. Front. Neurol..

[48] Lopes MA, Goodfellow M, Terry JR (2019). A model-based assessment of the seizure onset zone predictive power to inform the epileptogenic zone. Front. Comput. Neurosci..

