# Supplementary material for: Revealing epilepsy type using a computational analysis of interictal EEG
Source: Sci Rep. 2019 Jul 15;9:10169. doi: 10.1038/s41598-019-46633-7 (PMC6629665; doi:10.1038/s41598-019-46633-7)
Supplement: Supplementary file 1 — Supplementary Information [file 41598_2019_46633_MOESM1_ESM.pdf]

Supplementary Information

Revealing epilepsy type using a computational analysis of interictal EEG

Marinho A. Lopes[a,b,c,*], Suejen Perani[d], Siti N. Yaakub[d], Mark P. Richardson[c,d,e], Marc Goodfellow[a,b,c,#], John R. Terry[a,b,c,#]

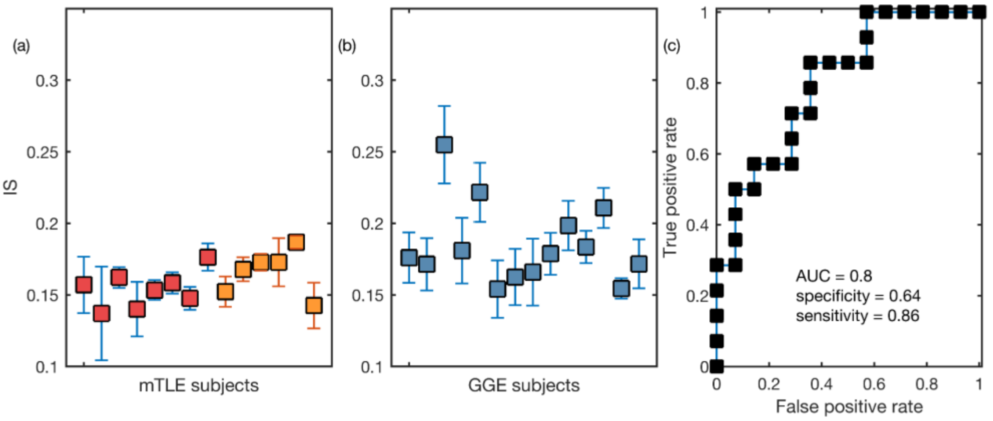

Supplementary Figure S1: Ictogenic Spread (IS) of the GGE and mTLE individuals. This figure is equivalent to figure 3, except that here we performed the comparison in a subset of individuals age and gender matched (age was tested using one-way analysis of variance (ANOVA), $p = 0.08$; and gender was assessed using Fisher's exact test, $p = 1$). Each marker in panels (a) and (b) represents the mean IS of a single individual and the error bars account for the variability of IS measured across different functional networks of different EEG segments band-pass filtered between 1 and 25 Hz. Panel (a) and (b) show the IS of mTLE and GGE subjects, respectively. In panel (a), the red markers identify left mTLE individuals, whist the orange markers correspond to right mTLE individuals. The GGE group has a larger IS than the mTLE group ($p = 0.004$, Mann–Whitney U test). Panel (c) exhibits the receiver operating characteristic (ROC) curve for GGE versus mTLE subjects using the IS as a classifier. The area under the curve (AUC) is 0.80, and the optimal specificity and sensitivity are 0.64 and 0.86, respectively.

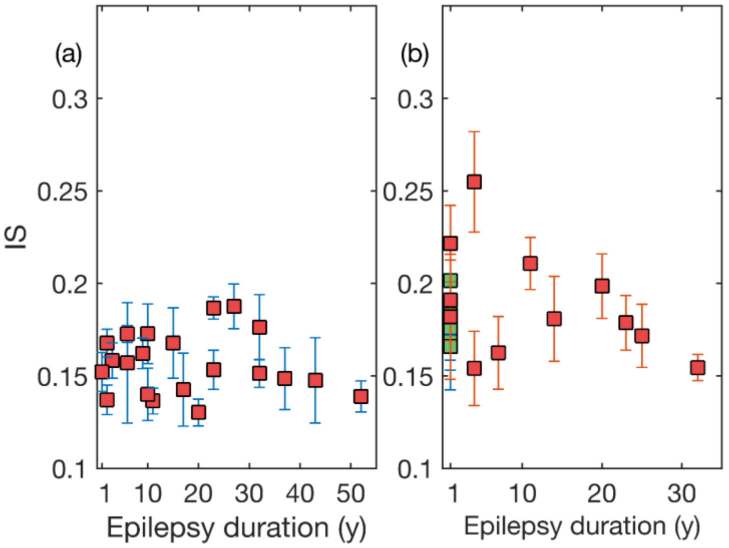

Supplementary Figure S2: Ictogenic spread (IS) as a function of epilepsy duration within (a) the mTLE group, and (b) the GGE group. Red markers correspond to non-seizure-free individuals, whilst green represent seizure-free individuals. There is no significant correlation or apparent dependence of

the IS on epilepsy duration (Pearson's correlation $|\rho| < 0.3$ in both groups). Also, IS of seizure-free individuals is not statistically different from IS measured in non-seizure-free individuals (Mann–Whitney U test, $p = 0.7$).

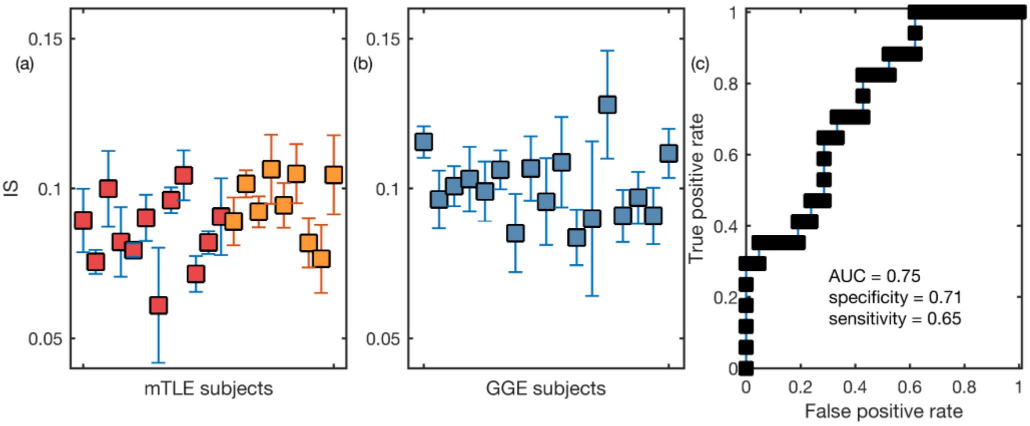

Supplementary Figure S3: Ictogenic Spread (IS) of the GGE and mTLE individuals. This figure is equivalent to figure 3, except that here we performed the comparison using only data from the standard clinical 19 channels. Each marker in panels (a) and (b) represents the mean IS of a single individual and the error bars account for the variability of IS measured across different functional networks of different EEG segments band-pass filtered between 1 and 25 Hz. Panel (a) and (b) show the IS of mTLE and GGE subjects, respectively. In panel (a), the red markers identify left mTLE individuals, whist the orange markers correspond to right mTLE individuals. The GGE group has a larger IS than the mTLE group ($p = 0.005$, Mann–Whitney U test). Panel (c) exhibits the receiver operating characteristic (ROC) curve for GGE versus mTLE subjects using the IS as a classifier. The area under the curve (AUC) is 0.75, and the optimal specificity and sensitivity are 0.71 and 0.65, respectively.
